# Antimicrobial Biodegradable Food Packaging Based on Chitosan and Metal/Metal-Oxide Bio-Nanocomposites: A Review

**DOI:** 10.3390/polym13162790

**Published:** 2021-08-19

**Authors:** Amin Babaei-Ghazvini, Bishnu Acharya, Darren R. Korber

**Affiliations:** 1 Department of Chemical and Biological Engineering, University of Saskatchewan, 57 Campus Drive, Saskatoon, SK S7N 5A9, Canada; amin.babaei@usask.ca; 2 Department of Food and Bioproduct Sciences, University of Saskatchewan, 51 Campus Drive, Saskatoon, SK S7N 5A8, Canada; darren.korber@usask.ca

**Keywords:** chitosan, antimicrobial, metallic nanomaterials, active packaging

## Abstract

Finding a practical alternative to decrease the use of conventional polymers in the plastic industry has become an acute concern since industrially-produced plastic waste, mainly conventional food packaging, has become an environmental crisis worldwide. Biodegradable polymers have attracted the attention of researchers as a possible alternative for fossil-based plastics. Chitosan-based packaging materials, in particular, have become a recent focus for the biodegradable food packaging sector due to their biodegradability, non-toxic nature, and antimicrobial properties. Chitosan, obtained from chitin, is the most abundant biopolymer in nature after cellulose. Chitosan is an ideal biomaterial for active packaging as it can be fabricated alone or combined with other polymers as well as metallic antimicrobial particles, either as layers or as coacervates for examination as functional components of active packaging systems. Chitosan-metal/metal oxide bio-nanocomposites have seen growing interest as antimicrobial packaging materials, with several different mechanisms of inhibition speculated to include direct physical interactions or chemical reactions (i.e., the production of reactive oxygen species as well as the increased dissolution of toxic metal cations). The use of chitosan and its metal/metal oxide (i.e., titanium dioxide, zinc oxide, and silver nanoparticles) bio-nanocomposites in packaging applications are the primary focus of discussion in this review.

## 1. Introduction

Plastic waste, especially classic food packaging, has become an environmental crisis around the world [1,2]. The global demand for biodegradable materials has motivated innovations in the plastics industry to develop polymers obtained from renewable bio-based resources [3,4,5]. Food packaging materials with acceptable mechanical properties [6], barrier properties [7], physical stability, recyclability, and biodegradability [8], as well as functional properties such as antimicrobial [9] and antioxidant activities [10], are highly desirable for food safety and for extending the shelf-life of packaged foods [11]. Currently, the majority of packaging materials produced are still dominated by conventional petroleum-based synthetic polymers since they are relatively cheap and processible with high reliability and durability [12,13,14]. While immediate changes to the packaging supply chain are incapable of replacing fossil fuel-based plastics at this time, significant advances have been made in the development of environmentally-friendly materials over the past several decades [15,16,17]. Carbohydrate-based polymers such as starch [18,19,20], chitosan [21,22,23,24], pullulan [25,26,27], and kefiran [28,29,30,31] have been the most considered biopolymers. The protein based polymers (i.e., whey protein [32], soy protein [27], and gelatin [33]) are now providing promising alternates to conventional non-degradable polymers.

Numerous biopolymer modification methods, including cross-linking by using ionizing rays (e.g., high energy UV-irradiation [19,29] and γ-irradiation [34]), magnetic fields [20], chemical reactions [35], the development of bio-nanocomposites [36], and combinations of two biopolymers [37] have also been recently evaluated to improve the functional properties of biopolymers.

Furthermore, the purpose of food packaging materials has more recently evolved from that of simple protection to a system that can provide a single or multiple functional roles in terms of food quality and preservation [38]. These roles could include modifying the inside of the package environment by either absorbing/adsorbing destructive molecules or compounds (such as oxygen and ethylene) [39], or releasing functional ingredients (such as antimicrobials and antioxidants or vitamins) [40]. With the employment of antimicrobial agents in food packaging materials, the growth of microbes could be prevented or delayed, with concurrent improvements in the shelf life of the enclosed foods [41]. In other words, antimicrobial packaging systems could release active agents continuously onto food surfaces, and thus provide extended inhibitory effects against targeted organisms [42]. This approach would improve the effectiveness of antimicrobials with respect to cost and function [21]. Hence, degradable antimicrobial packaging is a novel approach that offers multiple functionalities in a sustainable fashion, thereby addressing the current and future needs of the food sector. 

Antimicrobial compounds are generally classified into two main categories: (1) organic and inorganic materials and (2) natural materials. Some examples of organic antimicrobial agents include quaternary ammonium salts, halogenated compounds, organic acids, and phenols; natural materials are based on materials such as chitosan and chitin [22]. Inorganic antimicrobial agents, including metals and metal ions, metal oxide nanoparticles including TiO_2_ [43], ZnO [44], silver [45], gold [46], magnesium oxide [47,48] copper [49], copper oxide [50], iron (III) oxide [51], and CaO [52], have attracted considerable interest in food packaging researches due to their stability, especially under the different conditions imposed in food packaging [53,54].

Chitin is the second most abundant biopolymer on earth after cellulose, and it is found in the exoskeletons of crustaceans (e.g., crabs, shrimps, etc.) and insects [55,56,57]. Chitosan is a positively-charged, bio-based linear polysaccharide combined of randomly distributed β-(1-4)-linked D-glucosamine and N-acetyl-D-glucosamine units, and may be produced by the partial deacetylation of chitin. The obtained chitosan is soluble at lower pH solutions because it has amino groups which are basic and reacts with acidic compounds [58]. There are many amino groups on chitosan’s polymer chain, causing the molecule’s positive zeta potential. These amino functional groups have pKa’s around 6.5, and for this reason, at neural and lower pH systems, they tend to stay protonated [59]. Commercially-available chitosan is usually reported to have 80% and even up to 100% deacetylation [60] with molecular weights ranging from 35 to 800 kDa [61].

**Figure 1:**
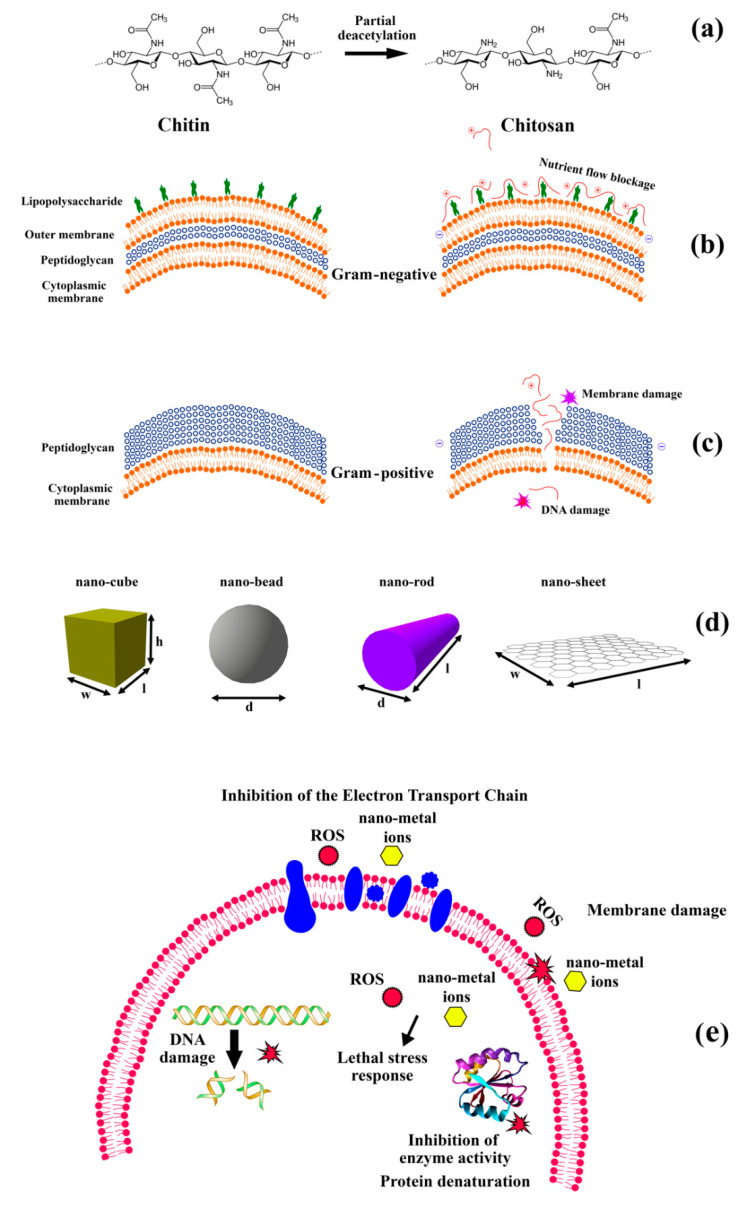
(**a**) Chitosan production by partial deacetylation of chitin, (**b**,**c**) schematic of the Gram-negative and -positive bacteria cell membranes and proposed models for the action of chitosan on cell membrane nutrient flow blockage and damage. The polycationic nature of chitosan causes the release of intercellular components, binding to bacterial DNA (inhibition of mRNA), blocking the nutrient flow and chelation of essential metals (redrawn from Kravanja, Primožič, Knez, and Leitgeb, 2019) [64], (**d**) schematic of different shapes of metal nanomaterials (reproduced from Cheeseman et al., 2020) [53] and (**e**) a summary of potential passive antimicrobial mechanisms of metal nanomaterials (not to scale) including physical interactions, release of ions, and production of ROS (adapted from Cheeseman et al., 2020) [53].

The mode of antimicrobial action of chitosan has not yet completely been established, and several “theories” exist. Figure 1 illustrates the chemical structure of the chitosan molecule and possible ways cationic polymers (such as chitosan and metallic nanoparticles) could induce membrane-level (and hence antimicrobial) effects on living cells. The most probable theory for chitosan’s lethal effects (Figure 1b,c) is that positively-charged amine groups (NH_3_^+^) of glucosamine interact with the negatively-charged outer membranes of bacteria, causing the formation of pores and resulting in leakage of intracellular components which ultimately causes cell death [62]. In addition to this, other possibilities, such as attachment of chitosan to DNA, could interfere with mRNA replication after chitosan penetrates the cytoplasmic membrane and crosses into the cytoplasm of microorganisms [63].

Regarding possible antimicrobial effects of metallic nanomaterials, several different mechanisms have been proposed and studied, including direct physical interactions or chemical reactions, the production of reactive oxygen species (ROS), and the increased dissolution of toxic metal cations [53,64,65] (Figure 1c–e).

This review will focus on recent major developments in the chitosan-based nanocomposites using titanium dioxide, zinc oxide, and silver nanomaterials. Since metallic nanoparticles have been considered as promising alternatives for conventional antimicrobial agents, their use with chitosan as a cationic bio-based antimicrobial polysaccharide would provide functional features relevant to the biodegradable packaging field.

## 2. Comparison of Different Chitosan and Chitosan-Metal Nanocomposites

### 2.1. Chitosan Titanium Dioxide Nanocomposites

Among the various metal oxide nanomaterials, titanium dioxide (TiO_2_) nanoparticles (TNPs), also known as titanium (IV) oxide, is the natural form of oxidized titanium [65] which are hydrophobic, biocompatible, and have photocatalytic properties, ultraviolet (UV) light absorbance, and excellent antimicrobial properties [65,66,67]. TNPs are employed in various fields such as electronics, cosmetics, wound healing, environmental pollution repair, and active food packaging [68]. TNPs also have photocatalytic activity, especially under exposure to UV light in the presence of water and oxygen. Under these conditions, ROS can be produced resulting in the production of hydroxyl and superoxide free radicals [69]. These free radicals react with inner cellular macromolecules or the cell membrane phospholipids and thereby produce serious damage of the cell membrane integrity as well as damage to DNA. However, a detailed description of the reaction mechanism of TNPs has not yet been discovered [43,70]. Many academics have studied chitosan/TNP bio-nanocomposites as potential antimicrobial food packaging material. Chitosan/TNP nanocomposites are in the interest of researchers due to the synergic effect of the antimicrobial properties of chitosan and TNP together. TNPs play a photocatalytic role by releasing ROS and chitosan as a cationic polymer leading to the damage of microorganisms’ membrane and delaying food spoilage.

Lin et al. (2015) developed chitosan-TNP hybrids with silver nanoparticles (AgNPs) through a photochemical reduction method. They used chitosan as a reducing agent to fabricate the final combined nanocomposite. The nanocomposite concentration for chitosan, TNPs, and AgNPs, were 10 mg/mL, 5 mg/mL, and 10 mg/mL, respectively. They then measured the inhibition zones using the disc diffusion method against a nontoxigenic *Escherichia coli (E. coli)* O157:H7 strain (ATCC 700728) as a representative Gram-negative pathogen surrogate. AgNP-chitosan and TNP-chitosan films were tested as comparator samples against the test cultures. AgNP-chitosan and TNP-chitosan films showed no inhibitory effect (zero diameter of inhibition) under the test conditions. However, TNP-AgNP-chitosan nanocomposites showed a significant inhibitory effect with 12.2 ± 0.7 mm diameter of inhibition zone, which showed a booster effect of hybrid TNPs and AgNPs with chitosan biopolymer [71].

The antimicrobial effect of chitosan-TNP nanocomposites have been studied against other organisms. For example, Zhang et al. (2017) evaluated chitosan-TNP nanocomposites for their antimicrobial activity under visible light by for food packaging applications. Their chitosan-TNP films possessed efficient antimicrobial activities against four tested strains (i.e., *E. coli*, *Staphylococcus aureus (S. aureus)*, *Candida albicans (C. albicans)*, and *Aspergillus niger (A. niger))*, with 100% inhibition seen after 12 h. During their antimicrobial tests, chitosan-TNP films (20 mm × 20 mm) were prepared and then 0.1 mL of microbial cell suspension (∼10^6^ CFU/mL) was spread on the test film samples held under controlled conditions (20 W daylight lamp visible light, 22 ± 2 °C temperature, and 50 ± 5% humidity). After 4 h, the microbial cells were washed (5×) from the films using 2 mL sterile 0.85% saline. Then, the number of viable cells remaining were enumerated using the spread plate count method [72].

The concentration of antimicrobial agents also plays an essential role in their inhibitory efficacy [73]. Different concentrations of TNPs could have different inhibition effects on microorganisms. Siripatrawan et al. (2018) developed active packaging from a combination of chitosan and TNPs over a range of concentrations (0, 0.25, 0.5, 1, and 2% *w*/*w*) and tested the produced composites for application as an ethylene scavenging system as well as an antimicrobial film. Based on tensile strength, water barrier, and ethylene photocatalytic degradation properties, chitosan film containing 1% TNPs (CT1) was optimal and therefore was selected for further evaluation for antimicrobial effects. They reported that CT1 exhibited antimicrobial activity against Gram-positive (*S. aureus*) and Gram-negative (*E. coli*, *Salmonella Typhimurium*, and *Pseudomonas aeruginosa*) bacteria and fungi (*Aspergillus* and *Penicillium*). Based on their work, chitosan-TNPs nanocomposite films are believed to have more broad application as an active packaging system for various postharvest food applications [74]. Detailed information of their results against different microorganisms is presented in Table 1.

Besides different concentrations of TNPs, mixing them with bio-based antimicrobial extracts offer “green” options for enhancing the antimicrobial properties of the films. Zhang et al. (2019) developed multifunctional food packaging films based on chitosan, TNPs and black plum peel extract (BPPE). They evaluated the antimicrobial activity of the produced films against four food pathogens, including *E. coli*, *S. aureus, Salmonella,* and *Listeria monocytogenes.* They reported that the chitosan-TNPs-BPPE composite film showed the greatest antimicrobial activity compared to the other samples, which probably was due to the synergistic/combined antimicrobial effect of chitosan, TiO_2_, and BPPE in the films. Additionally, the author’s study confirmed higher antimicrobial activity of their films against Gram-positive than Gram-negative bacteria, which they attributed to differences in the cell membrane structure [75].

In recent work by Hanafy et al. (2021), the authors produced a series of different combinations of thin chitosan-TNPs-oleic acid nanocomposite films formed using casting methods. The antimicrobial effects of the nanocomposite films were investigated by determining zones of inhibition against *B. cereus*, *S. aureus*, *C. albicans*, *A. niger*, and *E. coli.* The authors reported an increase in antimicrobial activity against *B. cereus*, *S. aureus*, and *A. niger* as a consequence of increasing the concentration of TiO_2_ to 15 wt%. A reverse trend for *C. albicans* was reported whereby increasing the amount of TNPs, the films showed less antimicrobial activity against *C. albicans* compared to pure chitosan [76]. Table 1 summarizes the antimicrobial property of chitosan-TNP on different microorganism.

As mentioned before, TNPs exhibit photocatalytic activity, especially under exposure to UV light in the presence of water and oxygen. Some researchers assessed TNPs antimicrobial performance under different lighting conditions. Qu et al. (2019) reported both improved mechanical and antimicrobial properties of a chitosan-zein films following the addition of highly dispersed TNPs. According to their report, the antibacterial effect was evaluated by measuring the diameter of the inhibition zone in dark and UV light (UV irradiation for 30 min), respectively. As in some other reports, the antibacterial effect of composite films against *S. aureus* (Gram-positive) was found to be greater than against *E. coli* or *S. enteritidis* (Gram-negative). Also, the antibacterial effect of UV light was greater than when incubated under dark conditions. In other words, the inhibition zone of the composite membrane against the three bacteria under UV irradiation was greater than under dark conditions, although no significant difference was found in antibacterial activity among the composite films with different TiO_2_ contents under dark condition for 24 h. This confirms that the TNPs have higher lethal effect under exposure to UV due to the photocatalytic activity and production of ROS in the medium [77].

Lastly, Lan et al. (2021) developed and studied multifunctional packaging films based on chitosan, nano-TiO_2_, and red apple pomace (APE). The diameter of inhibition zones was estimated for the different combination of the nanocomposite films against *E. coli* and *S. aureus*. Similar to other reports, all of the studied films had more effective antimicrobial activities against Gram-positive (*S. aureus*) compared with Gram-negative bacteria (*E. coli*) [78].

### 2.2. Chitosan-Zinc Oxide Nanocomposites

**Figure 2:**
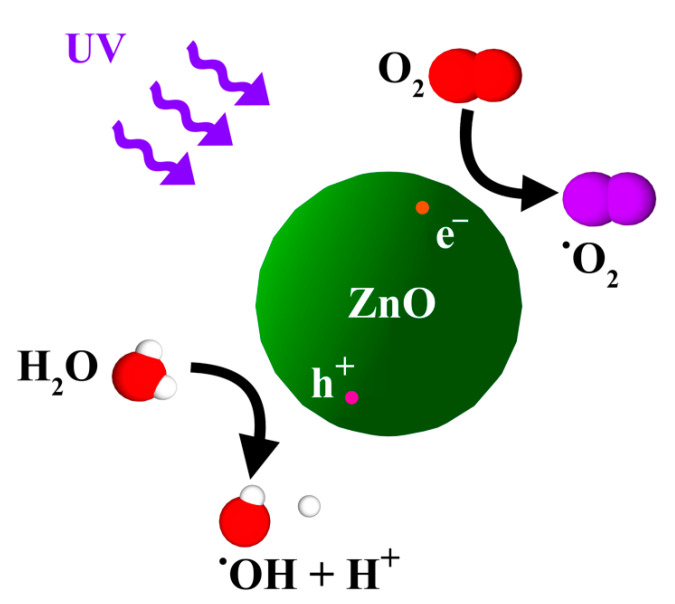
UV-induced photocatalytic activity of ZnO in the presence of O_2_ and H_2_O with consequent production of superoxide and hydroxyl radicals.

ZnO nanoparticles (ZNPs) are low-cost nanoparticles with unique catalytic, electrical (i.e., piezo- and pyro-electric) and optical, photostability, biocompatibility, biodegradability and, most importantly, antimicrobial properties [29]. For example, ZNPs were recently shown to possess high UV absorption, high photocatalytic efficiency, and higher biocompatibility than TNPs [79]. ZNPs in the presence of water and UV light can produce ROS that include hydrogen peroxide (H_2_O_2_) and superoxide. A brief description of the possible photochemical reactions that may occur with ZNP are shown in a series of equations (Equations (1)–(4)) and Figure 2. Upon UV irradiation, valence band electrons (e^−^) are promoted to the conduction band leaving a hole (h^+^) behind (Equation (1)). The holes at the ZNPs valence band can oxidize adsorbed water or hydroxide ions to produce hydroxyl radicals (Equation (2)). Electrons in the conduction band on the catalyst surface can reduce molecular oxygen to superoxide anion (Equation (3)). This radical may form organic peroxides or hydrogen peroxide in the presence of organic scavengers (Equation (4)). The hydroxyl radical is a powerful oxidizing agent and attacks organic macromolecules and compounds [29,80].

ZnO + hν → e^−^ + h^+^ (1)

h^+^ + H_2_O → •OH + H^+^ (2)

e^−^ + O_2_ → O_2_•^−^ (3)

O_2_•^−^ + HO_2_• + H^+^ → H_2_O_2_ + O_2_ (4)

Fabrication of well-dispersed and proper ZnO/chitosan nanocomposites is not straightforward due to the fact that chitosan is only soluble in an acidic environment. On the other hand, ZNPs under acidic condition switch into aqueous Zn^2+^ ions and result in production of chitosan-metal ion complexes and not nanocomposites [81]. Generally, to fabricate chitosan-ZnO nanocomposites, most researchers follow a two-step process. First, the addition of ZNPs in chitosan low-pH solution is used to produce chitosan-Zn ion complexes. Following that, the in situ crystallization of ε-Zn(OH)_2_ is accomplished using hot alkaline treatment that converts the complexes into chitosan-ZnO nanocomposites [82].

Youssef et al. (2015) prepared ZNP/AgNP-chitosan nanocomposites for food packaging applications. Accordingly, ZNPs were synthesized using the hydrothermal method, whereas AgNPs were prepared by a direct approach in the presence of chitosan. Furthermore, the authors investigated the effect of acid type (formic or acetic acid) on chitosan films as a dissolving agent. Chitosan-based nanocomposite films yielded good antimicrobial activity using the disc diffusion method against Gram-negative organisms (i.e., *E. coli*, *Salmonella Typhimurium* as well as Gram-positive (*S. aureus*, *B. cereus*, and *L. monocytogenes*) bacteria. Chitosan films dissolved in both acids showed significant inhibition zones against all tested strains. The addition of ZNPs and AgNPs increased the nanocomposites’ antimicrobial activity. An increase in the concentration of those nanoparticles increased the inhibition zone, with the largest inhibition zone being for a formic acid-dissolved chitosan ZNP nanocomposite, with the following diameters against test strains: *E. coli* (18 mm), *S. Typhimurium* (19 mm), *S. aureus* (15 mm), *B. cereus* (18 mm), and *L. monocytogenes* (16 mm) [83]. They did not study the hybrid application of ZNPs and AgNPs, which could be an interesting study to discover possible synergic antimicrobial effects of the nanoparticles.

Chitosan-ZNPs (ZNPs with a size of 35–45 nm) as an antimicrobial coating on polyethylene (PE) films has been studied by Al-Naamani et al. (2016). By employing an oxygen plasma pretreatment of PE films, the adhesion of the chitosan-ZNP nanocomposite coating layer on the PE surface increased by 2%. The antimicrobial effect of coated (with pure chitosan and chitosan-ZNPs nanocomposite) and un-coated films was evaluated by suspension culture medium (a suspension of test microorganisms on the film specimens). The films were tested against two Gram-negative bacteria, *E. coli* (ATCC 25922), *Salmonella enterica* serovar Typhimirium (ATCC 14028), and one Gram-positive bacteria, *S. aureus* (ATCC 6538). Their results showed that both chitosan-coated PE and chitosan-ZNP coated PE significantly inhibited bacterial growth. PE films did not show any antibacterial effect, as was expected. The chitosan coating had high antimicrobial activity against all tested bacteria with 1.3, 1.6, and 1.4 log reduction against *S. Typhimurium*, *E. coli*, and *S. aureus*, respectively. Alternatively, after 24 h of incubation, the complete growth inhibition resulted from antimicrobial tests using the chitosan-ZNP coatings, which the authors highlighted as having potential for industrial antimicrobial packaging uses [84]. Based on their study, PE coated with chitosan-ZNP nanocomposites offer a promising technique to enhance the antimicrobial properties of the PE films, which is one of the regular petroleum-based plastics used by the food packaging industry. With the reported method, it was possible to inactivate about 99.9% of pathogenic bacteria cells to increase the shelf life of food products and improve safety.

Different concentrations of ZNPs alone (and in combination with essential oils) have been studied to increase antimicrobial properties of ZNPs film. To increase the physical, mechanical, and antimicrobial properties of the chitosan-based nanocomposites, Sani et al. (2019) developed a chitosan-ZNPs film with Melissa essential oil. Their produced films contained ZNPs (0, 1, and 3% (*w*/*v*)) and Melissa essential oil (0, 0.25, and 0.5% (*w*/*v*)) as reinforcing agents, based on the initial chitosan solution’s dry matter concentration, to enhance the functional properties of the films. The disc diffusion method was used to evaluate the antimicrobial properties of the nanocomposites. The prepared film discs (diameter = 15 mm) were placed on agar plates containing a lawn of *E. coli* (ATCC 11775) bacteria. Based on their report, all of the films showed an inhibitory effect that was enhanced by the addition of ZNPs and essential oil. The highest inhibition zone was determined to be the chitosan-ZNP-Melissa essential oil composite preparation, which boosted the antimicrobial effect by the simultaneous use of ZNPs and essential oils [85].

Combining chitosan-ZnO nanocomposites with other biopolymers has been another approach used to increase the potential packaging properties of ZNP-chitosan bio-nanocomposites. In these studies, chitosan and gelatin nanocomposite hybrid films containing green-synthesized ZNPs were developed, and their properties studied by Kumar et al. (2020). The developed films with 2% and 4% ZNPs were again tested for antimicrobial properties using the disc diffusion method; the authors showed that the nanocomposite film had significant antimicrobial activity against *E*. *coli*. The zones of inhibition of the developed hybrid films containing 1%, 2%, and 4% ZNPs were 10.5, 10.5, and 10.7 mm in diameter against *E. coli*, respectively [86].

Ahmed et al. (2021) compared nanocomposites films containing chitosan nanoparticles as an organic filler and ZNPs as an inorganic filler to evaluate their different reinforcement method in gelatin/tapioca starch films. They also studied the antimicrobial effect of the films using zones of inhibition against Gram-negative bacteria (*E. coli*) and Gram-positive bacteria (*S. aureus*) [87].

In yet another polymer blending approach, Boura-Theodoridou et al. (2020) investigated the performance of chitosan-ZnO nanocomposite film for antimicrobial packaging applications as a function of NaOH treatment and glycerol/poly (vinyl alcohol) proportions. They reported the successful formation and growth of ZnO nanoparticles in chitosan-based films following immersion in hot NaOH solution. Antibacterial activity of the nanocomposites was studied against a panel of organisms that included an *E. coli* (Gram-negative) isolate, along with *B. lactofermentum* and *C. glutamicum* (Gram-positive) strains. Pure chitosan films resulted in almost 100% inhibition of the growth of all three bacteria. However, they reported that the effect of ZNP content was not clear. They reported a general reduction of antimicrobial efficacy due to the immersion of the films in NaOH solution which lowers the polycationic character and the solubility of chitosan. In general, the antimicrobial activity of the produced chitosan-ZNPs nanocomposites was greatest against *B. lactofermentum*, moderate against *E. coli*, and almost absent against *C. glutamicum* [88]. This suggests the inhibition ability of the pristine chitosan films against *C. glutamicum* and *E. coli* bacteria before and after growth of ZNPs in the nanocomposite structure is different due to the decrease in polycationic nature of the chitosan caused by the alkaline treatment.

Preparation of multilayer films consisting of chitosan, sodium alginate, and carboxymethyl chitosan-ZnO nanoparticles was studied by Wang et al. (2019). Their study demonstrated distinct antibacterial activities against *S. aureus* and *E. coli*, and that a significant positive correlation existed between percentage of ZNPs and film antibacterial efficacy [89]. Qui et al. (2019) developed flexible chitosan-ZNP nanocomposite films by in situ precipitation of ZNPs in a chitosan matrix with an alkaline treatment. Accordingly, they reported their chitosan-ZNP films caused 3.4-log and 4.0-log reductions in viable *E. coli* and *S. aureus* cells after 0.5 h exposure, respectively [90]. Akhil Krishnan et al. (2020) produced chitosan-ZNP nanocomposites which the ZNPs were synthesized using orange peel oil using a “green” chemical reduction method. Antibacterial activity of the films investigated by the agar disc diffusion method against *E. coli,* wherein a distinct inhibition of the microorganisms for the ZNP-loaded films was observed. According to the report, the chitosan-ZNP films showed 1.9 ± 0.1 cm zone of inhibition, while pristine chitosan film represented a zone of inhibition of 0.9 ± 0.1 cm [91]. Yadav et al. (2021) developed food packaging materials based on chitosan and ZNP-loaded gallic acid with improved antibacterial properties against both Gram positive (*B. subtilis*) and Gram negative (*E. coli*) bacteria. Reduced antibacterial activity was seen with pure chitosan in comparison to the films dosed with ZNPs and gallic acid in the matrix. It was revealed that increasing ZNPs and gallic acid concentrations from 30 to 70 mg in the film matrix caused significant increases in antibacterial activity [23].

The effect of size of the ZNPs on antimicrobial properties in chitosan matrix were also studied by Zhang et al. (2021). In their work, chitosan nanocomposite films were prepared by incorporating different sizes of zinc oxide particles of 5 μm, 50 nm, and 100 nm. Antimicrobial activity of the films against *E. coli* and *S. aureus* revealed that films containing 0.3% of 50 nm zinc oxide particles exhibited the best extent of inhibition. Their result showed the size-dependent activity of ZNPs, with smaller ZNPs having enhanced antibacterial activities [92]. A summary of the recent works with more details has been represented in Table 2.

### 2.3. Chitosan-Silver Nanocomposites

Silver nanoparticles (AgNPs) are defined in the literature as compounds containing a large percentage of silver oxide due to the high ratio of silver atoms in the bulk surface. Generally, silver salt and silver-based materials have been well known for their antimicrobial properties since ancient times [93]. Several antimicrobial mechanisms for AgNP activity have been reported, including production and release of Ag ^+^ ions, ROS generation in the outer and inner membrane of microorganisms, cell membrane interference, ribosome destabilization, and mitochondrial and nucleic acids damage (Figure 1e) [94]. The synthesis of Ag-NPs can be achieved by physical, chemical, and biological methods. The biological method for Ag-NPs production could be considered to be an environmentally-friendly process, whereas other physical and chemical methods use high amounts of energy and chemical solvents, which are considered toxic, resulting in restrictions on the synthesized nanoparticles potential biomedical and food application [95,96]. 

Qin et al. (2019) developed chitosan-based active and intelligent food packaging films with incorporated AgNPs (2%) and purple corn extract. The antibacterial performance of the produced films was studied against *E. coli*, *Salmonella*, *S. aureus*, and *L. monocytogenes*. Based on their report, chitosan nanocomposites reinforced with AgNPs showed five times higher antimicrobial effects against four foodborne pathogens compared to pure chitosan films [97]. An antioxidant and antibacterial chitosan-tea polyphenols-silver nanoparticle composite films were developed via a novel one-pot method by Zhang and Jiang (2020). Accordingly, AgNPs were produced by reducing AgNO_3_ using 0.1% (*w*/*v*) tea polyphenol solution. The produced nanocomposites were investigated for their antibacterial performance against *S. aureus* and *E. coli.* All of the films with AgNPs presented stronger antibacterial activity against Gram-negative than Gram-positive bacteria [98].

To overcome the drawbacks of pure chitosan films, Cao et al. (2020) developed a new combination of films containing catechol-modified chitosan, AgNPs, and gelatin. The prepared bio-nanocomposites showed exceptional antibacterial behaviors against *S. aureus* and *E. coli* with up to 65% and 70% bacterial death percentage, respectively. Their results also confirmed a higher antibacterial behavior against Gram-negative than Gram-positive bacteria [99]. In another study, Kadam et al., (2019), the pH-dependent sustained release of AgNPs synthesized using *Nigella sativa* extract with a biogenic method from chitosan matrix was demonstrated. The antibacterial performance of the chitosan-based nanocomposite films was investigated against the Gram-positive bacteria *S. aureus* and *B. subtilis* and Gram-negative bacteria *P. aeruginosa* and *E. coli* using a disc diffusion inhibition method. According to their results, composite films demonstrated better antibacterial activity against Gram-negative bacteria compared to the Gram-positive bacteria, which confirms findings seen in similar studies. Additionally, smaller sized AgNPs (8 nm) showed greater lethal effects against the studied bacteria [100].

Pandey et al. (2020) developed chitosan-AgNP nanocomposites for food packaging applications. They fabricated the nanocomposite with AgNPs sizes of 80 ± 11 nm in chitosan in a polyvinyl alcohol (PVA) blend to form electrospun fibrous composite nano-layers. The antimicrobial activity of the fibrous layer was then analyzed with the agar disc diffusion against *E. coli* and *L. monocytogenes* bacteria. The PVA nano-layer did not show any inhibition effect, which was expected. However, the PVA-chitosan and AgNP nano-layers showed a remarkable inhibitory effect against both tested strains. The maximum zone of inhibition (20 mm for *E. coli*, and 21 mm for *L. monocytogenes*) was observed for PVA (70%)-chitosan (30%)-AgNP nano-layers, possibly due to the synergistic antimicrobial activity of chitosan and AgNPs. Also, PVA (70%)-chitosan (30%) nano-layers without AgNPs showed inhibition zones (16 mm for *E. coli*, and 15 mm for *L. monocytogenes*) due to the electrostatic interaction between the cationic chitosan molecule and negatively charged bacterial cell membranes [101].

A recent study investigated the in vitro antifungal activity of two different chitosan (commercial and shrimp) AgNPs (100 to 250 nm diameter) nanocomposites [102]. The antifungal evaluation of the films against the phytopathogen *Botrytis cinerea* was studied since it is considered one of the most important postharvest pathogens in fruit and vegetables. The droplets of conidial solution were inoculated onto the films in a chamber, with a concentration of 10^6^ conidia per mL. An inhibition percentage of greater than 97% were reported by the authors for this study against this fungal organism.

Ghasemzadeh et al. (2021) developed a series of novel full polysaccharide chitosan-agarose-AgNPs nanocomposites with in situ reduction of silver ions in the polymeric network. The antimicrobial behavior of the films was evaluated against a panel of bacteria, including *P*. *aeruginosa*, *E*. *coli*, and *S*. *aureus*. Chitosan-agarose films did not show antibacterial activity using the disc diffusion method. However, the films containing AgNPs showed significant zones of inhibition against *S*. *aureus*, *E*. *coli*, and *P*. *aeruginosa* [103].

More recently, the effect of chitosan-essential oils-AgNP nanocomposite films on the shelf life of strawberries was examined by Shankar et al. (2021), with films showing strong antimicrobial activity against pathogenic bacteria (*E. coli*, *L. monocytogenes*, *Salmonella*) as well as a fungal strain (*A. niger*). The chitosan-AgNP nanocomposites reduced *E. coli* viable counts by 3.4-log CFU/g, *L. monocytogenes* by 3.0-log CFU/g, *Salmonella* by 1.4-log CFU/g, and *A. niger* by 0.5-log CFU/g after 16 h exposure [104].

In an interesting novel food packaging approach, Ramadan et al. (2020) developed cotton fabrics dipped in chitosan solution, dried, and then loaded with silver nanoparticles by an *in situ* technique. Their research introduced a potential new use of chitosan and metallic nanoparticles for dried food packaging. These antimicrobial fabrics could see use in various packaging processes including the packaging of seeds and powder materials. The inhibitory effects of treated fabrics against Gram negative (*P. aeruginosa*) and Gram positive (*S. aureus*) bacteria, fungi (*A. niger*) and yeast (*C. albicans*) were investigated. Overall, the fabrics loaded with chitosan and AgNPs showed good antimicrobial properties using a disc diffusion method [105].

## 3. Discussion and Conclusions

Plastic packaging or chemical additives are two primary methods used by the food industry to protect and prevent their products from spoilage. The side effects of these methods on human health are well-known, including direct chemical additives to the food or chemical migration from packaging into the food content [106,107]. Researchers have extensively studied the possible alternative compounds such as polymers and nanomaterials to address these problems. Chitosan and its derivatives seem to be promising biodegradable and biocompatible polymers for food packaging; however, they still suffer from a lack of good packaging properties (e.g., mechanical, thermal, and hydrophobic properties). Hence, the metallic and metal oxide nanomaterials (e.g., TiO_2_, ZnO, and Ag) were employed to reinforce chitosan-based materials and give them functional properties, including antimicrobial activity. Researchers are still evaluating the respective benefits of these classes of nanomaterials. Based on the current review of the recent reports on using these nanomaterials with chitosan as nanocomposites, the following conclusions of their antimicrobial effects were compiled:

- Chitosan has a great film-forming ability, making this biopolymer a suitable candidate for biodegradable food packaging research. Most of the chitosan-based bio-nanocomposites have been fabricated through the water-based solution casting method. Due to chitosan’s cationic nature, it is soluble in water under acidic conditions (1–2% acetic or formic acid solution). Chitosan films obtained through this method have a lower final pH because of the organic acid in their structure, a feature which could cause bolster inhibition of some microorganisms such as coliforms.
- TiO_2_ nanoparticles have photocatalytic activity, especially under exposure to UV light in the presence of water and oxygen. Under these conditions, ROS can be generated, resulting in production of hydroxyl and superoxide free radicals. These free radicals react with inner cellular macromolecules or the cell membrane phospholipids resulting in severe damage to cell membrane integrity as well as DNA. However, a detailed description of the reaction mechanism of TNPs has not yet been discovered. Reports on TiO_2_ and chitosan nanocomposites showed more significant inhibition as compared to pure chitosan films due to the additional inhibitory effect of TiO_2_ nanoparticles. TiO_2_ nanoparticles show a more significant inhibitory effect under UV exposure comparing white light or dark storage. More antimicrobial activities against Gram-positive compared with Gram-negative bacteria with TiO_2_ nanocomposites have been reported. As mentioned above, the lower pH has a booster effect on the inhibition of chitosan-based nanocomposites on some microorganisms.
- Like TiO_2_ nanoparticles, ZnO nanoparticles have photocatalytic activity but with higher biocompatibility and UV light absorption compared with TiO_2_. The antimicrobial activity of ZnO nanoparticles is similar to TiO_2_ nanoparticles in terms of ROS generation. However, the probable generation of hydrogen peroxide (H_2_O_2_) during the ZnO photocatalytic activity under UV-irradiation has been reported. Fabrication of chitosan with ZnO nanocomposites is different than TiO_2_/chitosan nanocomposites because ZnO nanoparticles may release aqueous Zn^2+^, which changes the morphology and efficacy of the nanoparticles. Hence, in situ crystallization of ε-Zn (OH)_2_ using hot alkaline treatment converts the complexes into chitosan-ZnO nanocomposites is a common method to fabricate ZnO chitosan nanocomposites. However, hot alkaline treatment might cause some changes in chitosan polycationic nature, which could be considered a drawback of this method. There has been no report to date on different antimicrobial efficacies against Gram-negative and Gram-positive for chitosan-ZnO nanocomposites. Also, greater inhibitory effects of chitosan-ZnO and Ag hybrid nanocomposites has also been reported.
- Silver salt and silver-based materials have been well known for their antimicrobial properties since ancient times. Release of Ag ^+^ ions, ROS generation, and cell membrane disruption are the most cited possible antimicrobial mechanisms linked with AgNPs. Works that studied chitosan-AgNPs films reported more potent antibacterial activity against Gram-negative than Gram-positive bacteria, which could be an advantage in combination with other nanoparticles with greater antimicrobial activities against Gram-positive compared. Also, the effect of particle size on the efficacy of the studied nanomaterials showed a smaller range of nanoparticle size offers a more significant inhibition effect against microorganisms. However, the regulations regarding their use in the food industry are still changing and need to be studied more. For example, on a recent announcement from the European Food and Safety Authority (EFSA), the use of TiO_2_ is no longer considered safe [108].
- Due to the biodegradability, sustainability, and effective film forming properties of chitosan, it has been a promising polysaccharide of interest for researchers in the field of food packaging. The cationic nature of the chitosan shows antimicrobial properties, but not sufficient by itself to be used as the sole antimicrobial agent. Among many metals and metal oxide options, TiO_2_, ZnO, and Ag are ideal nanomaterials to be employed in conjunction with chitosan to enhance the antimicrobial performance of the final films. However, as mentioned above, the cytotoxicity of these materials is still unknown and thus further studies are needed for application in the food industry.

## Figures and Tables

**Table 1:** Chitosan-TNP films antimicrobial activity.

Biopolymer Films	Concentration of TNPs	Tested Microbe	Results	Reference
Chitosan-AgNPs-TNPs	5 mg/mL	*Escherichia coli* O157:H7	TNPs: 7.3 × 109 CFU mL^−1^ Chitosan: 4.0 × 109 CFU mL^−1^ Chitosan-TNPs: 5.6 × 109 CFU mL^−1^ Chitosan-TNPs-AgNPs 3.2 × 103 CFU mL^−1^	[71]
Chitosan-TNPs	N/A	*E. coli*	100% sterilization in 12 h on all of the tested microorganisms	[72]
		*S. aureus*		
		*C. albicans*		
		*A. niger*		
Chitosan-TNPs Chitosan-TNPs + UV treated	1%	*E. coli*	NUV: 27%, UV: 53%	[74]
		*S. aureus*	NUV: 38%, UV: 53%	
		*S. Typhimurium*	NUV: 11%, UV: 22%	
		*P. aeruginosa*	NUV: 12%, UV: 15%	
		*Aspergillus*	NUV: 17%, UV: 21%	
		*Penicillium*	NUV: 5%, UV: 8%	
Chitosan-TNPs	0 wt%	*B. cereus*	70.14 ± 0.02 (%)	[76]
		*S. aureus*	81.49 ± 0.20 (%)	
		*C. albicans*	72.46 ± 0.08 (%)	
		*A. niger*	54.25 ± 0.02 (%)	
		*E. coli*	55.11 ± 0.03 (%)	
	2 wt% based on chitosan solution	*B. cereus*	75.50 ± 0.15 (%)	
		*S. aureus*	82.45 ± 0.04 (%)	
		*C. albicans*	76.36 ± 0.31 (%)	
		*A. niger*	86.19 ± 0.25 (%)	
		*E. coli*	60.00 ± 0.04 (%)	
	5 wt% based on chitosan solution	*B. cereus*	78.87 ± 0.05 (%)	
		*S. aureus*	82.99 ± 0.18 (%)	
		*C. albicans*	62.55 ± 0.07 (%)	
		*A. niger*	88.49 ± 0.27 (%)	
		*E. coli*	57.45 ± 0.11 (%)	
	10 wt% based on chitosan solution	*B. cereus*	79.47 ± 0.01 (%)	
		*S. aureus*	82.25 ± 0.14 (%)	
		*C. albicans*	51.64 ± 0.06 (%)	
		*A. niger*	90.99 ± 0.21 (%)	
		*E. coli*	72.77 ± 0.15 (%)	
	15 wt% based on chitosan solution	*B. cereus*	85.85 ± 0.21 (%)	
		*S. aureus*	84.62 ± 0.18 (%)	
		*C. albicans*	44.82 ± 0.09 (%)	
		*A. niger*	93.99 ± 0.29 (%)	
		*E. coli*	60.00 ± 0.25 (%)	
Chitosan-TNPs-oleic acid	15 wt% based on chitosan solution	*B. cereus*	50.70 ± 0.24 (%)	
		*S. aureus*	82.21 ± 0.22 (%)	
		*C. albicans*	71.27 ± 0.17 (%)	
		*A. niger*	95.50 ± 0.28 (%)	
		*E. coli*	42.55 ± 0.10 (%)	

(%): Growth Inhibition Percentage, UV and NUV: UV and non-UV treated.

**Table 2:** Chitosan-ZNP films antimicrobial activity.

Biopolymer Films	Concentration of ZNPs		Tested Microbe	Results	Reference
Chitosan-ZNPs (Formic and acetic acid treated)	N/A		*E. coli*	F: 18 mm A: 15 mm	[83]
			*S. Typhimurium*	F: 19 mm A: 17 mm	
			*S. aureus*	F: 15 mm A: 13 mm	
			*B. cereus*	F: 18 mm A: 13 mm	
			*L. monocytogenes*	F: 16 mm A: 10 mm	
Chitosan-AgNPs (Formic and acetic acid treated)	N/A		*E. coli*	F: 12 mm A: 14 mm	
			*S. Typhimurium*	F: 15 mm A: 13 mm	
			*S. aureus*	F: 10 mm A: 11 mm	
			*B. cereus*	F: 12 mm A: 12 mm	
			*L. monocytogenes*	F: 11 mm A: 10 mm	
Chitosan-ZNPs	**ZNPs size (nm)**	0 wt%	*B. lactofermentum*	≈100 (%)	[88]
			*C. glutamicum*	≈100 (%)	
	N/A		*E. coli*	≈100 (%)	
	6.9	3 wt%	*B. lactofermentum*	≈60 (%)	
			*C. glutamicum*	≈10 (%)	
			*E. coli*	≈60 (%)	
	9.3	5 wt%	*B. lactofermentum*	≈100 (%)	
			*C. glutamicum*	≈7 (%)	
			*E. coli*	≈30 (%)	
	14.6	7 wt%	*B. lactofermentum*	≈90 (%)	
			*C. glutamicum*	≈5 (%)	
			*E. coli*	≈40 (%)	
Chitosan-Gly-ZNPs	**ZNPs size (nm)**	0 wt%	*B. lactofermentum*	≈100 (%)	
			*C. glutamicum*	≈100 (%)	
	N/A		*E. coli*	≈100 (%)	
	7.5	3 wt%	*B. lactofermentum*	≈95 (%)	
			*C. glutamicum*	≈5 (%)	
			*E. coli*	≈30 (%)	
	10.8	5 wt%	*B. lactofermentum*	≈100 (%)	
			*C. glutamicum*	≈10 (%)	
			*E. coli*	≈50 (%)	
	14.8	7 wt%	*B. lactofermentum*	≈100 (%)	
			*C. glutamicum*	≈12 (%)	
			*E. coli*	≈15 (%)	
gelatin/tapioca starch-chitosan	12.5%		*E. coli*	45.29 ± 8.62 mm^2^	[87]
			*S. aureus*	45.29 ± 8.62 mm^2^	
gelatin/tapioca starch-ZNPs	12.5%		*E. coli*	85.30 ± 18.90 mm^2^	
			*S. aureus*	67.28 ± 17.28 mm^2^	

(mm): Inhibition zone diameter, F and A: formic and acetic acid included, (%): Inhibition growth percentage, Gly: Glycerol, (mm^2^): zone on inhibition (area).

## Data Availability

No data was reported.
